# A Set of Reliable Samples for the Study of Biomarkers for the Early Diagnosis of Parkinson's Disease

**DOI:** 10.3389/fneur.2022.844841

**Published:** 2022-05-30

**Authors:** Marcela Konjevod, Jorge Sáiz, Coral Barbas, Alberto Bergareche, Eva Ardanaz, José Ma Huerta, Ana Vinagre-Aragón, Ma Elena Erro, Ma Dolores Chirlaque, Eunate Abilleira, Jesús Ma Ibarluzea, Pilar Amiano

**Affiliations:** ^1^Division of Molecular Medicine, Rudjer Boskovic Institute, Zagreb, Croatia; ^2^Facultad de Farmacia, Centro de Metabolómica y Bioanálisis, Universidad San Pablo-CEU, CEU Universities, Madrid, Spain; ^3^Department of Neurology, University Hospital Donostia, San Sebastián, Spain; ^4^Neuroscience Area, Biodonostia Health Research Institute, San Sebastián, Spain; ^5^Biomedical Research Networking Centre Consortium for the Area of Neurodegenerative Diseases (CIBERNED), Instituto de Salud Carlos III, Madrid, Spain; ^6^Navarra Public Health Institute, Pamplona, Spain; ^7^Navarra Institute for Health Research (IdiSNA), Pamplona, Spain; ^8^Spanish Consortium for Research on Epidemiology and Public Health (CIBERESP), Instituto de Salud Carlos III, Madrid, Spain; ^9^Instituto Murciano de Investigación Biosanitaria, Murcia, Spain; ^10^Department of Neurology, Navarra Hospital Complex, Pamplona, Spain; ^11^Ministry of Health of the Basque Government, Public Health Laboratory in Gipuzkoa, San Sebastián, Spain; ^12^Epidemiology of Chronic and Comunnicable Diseases Area, Biodonostia Health Research Institute, San Sebastián, Spain; ^13^Ministry of Health of the Basque Government, Sub Directorate for Public Health and Addictions of Gipuzkoa, San Sebastián, Spain; ^14^Environmental Epidemiology and Child Development Area, Biodonostia Health Research Institute, San Sebastián, Spain; ^15^Faculty of Psychology, University of the Basque Country UPV/EHU, San Sebastian, Spain

**Keywords:** Parkinson's disease, biomarkers, metabolomics, fatty acids, EPIC-Navarra

## Abstract

**Background:**

Parkinson's disease (PD) is a progressive neurodegenerative disorder, diagnosed according to the clinical criteria that occur in already advanced stages of PD. The definition of biomarkers for the early diagnosis of PD represents a challenge that might improve treatment and avoid complications in this disease. Therefore, we propose a set of reliable samples for the identification of altered metabolites to find potential prognostic biomarkers for early PD.

**Methods:**

This case–control study included plasma samples of 12 patients with PD and 21 control subjects, from the Spanish European Prospective Investigation into Cancer and Nutrition (EPIC)-Navarra cohort, part of the EPIC-Spain study. All the case samples were provided by healthy volunteers who were followed-up for 15.9 (±4.1) years and developed PD disease later on, after the sample collection. Liquid chromatography coupled to tandem mass spectrometry was used for the analysis of samples.

**Results:**

Out of 40 that were selected and studied due to their involvement in established cases of PD, seven significantly different metabolites between PD cases and healthy control subjects were obtained in this study (benzoic acid, palmitic acid, oleic acid, stearic acid, myo-inositol, sorbitol, and quinolinic acid). These metabolites are related to mitochondrial dysfunction, the oxidative stress, and the mechanisms of energy production.

**Conclusion:**

We propose the samples from the EPIC study as reliable and invaluable samples for the search of early biomarkers of PD. Likewise, this study might also be a starting point in the establishment of a well-founded panel of metabolites that can be used for the early detection of this disease.

## Introduction

Metabolomics is an important well-established tool, able to provide useful insights of unknown biochemical mechanisms and possible biomarkers for various disorders (1). Understanding altered metabolic pathways and metabolites provides a better knowledge of underlying biological alterations. This information might improve the treatment strategies and define novel therapeutic approaches, as well as facilitate disease prediction and diagnosis. The metabolic profiles for neurodegenerative and neuropsychiatric disorders and the related metabolic pathways are still unclear (2, 3), as it is the case for Parkinson's disease (PD). However, some studies have shown an association between certain metabolites and several metabolic pathways in PD (2–4). Alterations in the tryptophan and kynurenine metabolism have been associated with the appearance of psychiatric symptoms and the development of PD. Certain metabolites, as part of this metabolic pathway, showed decreased levels in several biological fluids, such as tryptophan (4, 5), kynurenic acid (KA) (5, 6), and quinolinic acid (QA) (5, 6), while kynurenine (3), 5-hydroxytryptophan (3), xanthurenic acid (3), and 3-hydroxykynurenine (7) showed an elevation in PD. Due to the significance of this metabolic pathway, it is assumed that disruption of tryptophan and kynurenine metabolism might lead to neurotoxicity associated to PD (8). Dopamine and norepinephrine metabolism play an important role in PD development and progression. It is known that dopaminergic loss in the *substantia nigra* is one of the biggest hallmarks of PD (4, 5), while metabolites of the tricarboxylic acid cycle (TCA) might be associated with dopaminergic loss due to mitochondrial dysfunction and alterations in energy production (5). Several metabolites associated to these metabolic pathways have been observed to be altered in PD (5–9). Sugars and their derivatives (4–11) showed increased levels, while amino acids and their derivatives were altered in subjects with PD (4, 5, 12, 13). Changes in branched-chain amino acids might also implicate changes in protein synthesis, mitochondrial biogenesis, and autophagy (4). These findings largely indicate the involvement of mitochondrial dysfunction in PD pathogenesis. Furthermore, metabolites involved in fatty acid and lipid metabolisms, such as fatty acids, glycerophospholipids, carnitines, and bile acids, showed altered levels in subjects with PD, implying on the potential involvement of inflammation, increased rate of oxidative stress, impaired brain metabolism, and mitochondrial dysfunction in PD pathogenesis (2, 4, 14–17). Fatty acids mostly show reduced levels in patients with PD, possibly due to their vulnerability to the oxidative stress that causes lipid peroxidation and structural damage of fatty acids (18). Among other fatty acids, stearic acid (4, 5), oleic acid (4, 5), and palmitic acid (4, 5) showed significant changes in subjects with PD, compared to control subjects. Moreover, it has been reported that uric acid, together with other metabolites of purine metabolism, has a protective role against cell death and damage caused by oxidative stress (4, 5, 19). It is observed that people with lower levels of uric acid in the brain, serum, or plasma have higher risks to develop PD. Therefore, low levels of uric acid might be a potential biomarker for the early diagnosis of PD (19–21). In addition, other compounds, including alcohols, hydroxy acids, and amines (5, 9, 22, 23), have also been altered in patients with PD. However, the mechanisms that lead to these changes are still unclear.

It is noteworthy that these metabolic pathways have been related to PD in subjects with an already established disorder, meaning that these metabolites have been found in patients who had already developed PD at the time when those studies were carried out. Unsurprisingly, for PD as well as for other neurodegenerative disorders, the early diagnosis of the disorder is the greatest challenge. The main point is to detect the disorder before the neurodegeneration starts to proceed with adequate early treatment. In this regard, the scientists have not been able to find a set of metabolites that are altered before the disease is established. The complication of finding biomarkers for detecting disease before it emerges is to find reliable samples to be used. The studies published so far are mainly focused on biomarkers for the early stages of PD (24–26). The difficulty of these studies relies on the identification of patients who have recently developed PD, in the earliest possible stage.

The plasma samples used in this study were obtained from the European Prospective Investigation into Cancer and Nutrition (EPIC), a prospective, multicenter, cohort study. This study focuses on investigating the effects of several factors and the incidence of cancer and other chronic diseases, more than half a million participants from 23 different centers and 10 European countries were recruited and followed-up for almost 15 years. It is possible to study the bank sample to find those donors that were not diagnosed for a particular disease at the time of sample collection and developed the disease afterward during the monitoring period of this study. We used a subset of these samples from healthy participants (not diagnosed of PD or showing any PD-related symptoms) at baseline that were researched by expert epidemiologists to identify donors who had not a diagnosed PD and did not show any PD-related symptoms at the time of sample collection, but who developed PD later on. These samples are, therefore, of an extraordinary value for studying biomarkers that are altered before PD shows any symptoms and the neurodegeneration begins. Counting on those samples, we considered that investigating those metabolites that are known to be altered when PD is established can be a good starting point for finding biomarkers for the early diagnosis of the disease.

Therefore, this article aims to study the EPIC samples using liquid chromatography and mass spectrometry methodologies for finding metabolites that can be used as early biomarkers of PD. It is based on the ability of the EPIC samples to reveal early biomarkers for PD, considering the unique nature of those samples.

## Materials and Methods

### Subject Recruitment

This study was conducted at the Center for Metabolomics and Bioanalysis (CEMBIO) in Madrid, Spain. This case–control study included plasma samples from one Spanish cohort (EPIC-Navarra), part of the EPIC study. Biological samples, including plasma, serum, erythrocytes, and leukocytes, were collected and stored in liquid nitrogen. The exclusion criteria included physically or mentally incapability to participate in this study, as well as pregnancy and lactation for women. All the participants were free of cancer at the time of diagnosis. Detailed collected data about dietary intake and lifestyle of participants have been published previously. Sociodemographic characteristics and anthropometric measures were collected from all the participants using standard procedures (27). This study included 12 case plasma samples from subjects who had developed Parkinson's disease from the sampling time to June 2011 and 21 corresponding control samples. Both the groups consisted of 18.2% women, aged between 46 and 63 years old, and 81.8% men aged between 51 and 64 years old. Incident PD cases were ascertained by record linkage with health databases to identify potential cases, followed by individual revision of the medical history by expert neurologists in order to establish the diagnosis based on the available clinical records (28). Diagnosis of Parkinson's disease was established for participants fulfilling at least two of the following criteria: (1) primary care records using either codes 332 of the International Classification of Diseases-9 (ICD-9) or codes N87 of the International Classification of Primary Care; (2) registration of prescriptions, including subjects with at least one prescription of any of the N04-antiparkinsonian drugs of the Anatomical Therapeutic Chemical/Defined Daily Dose (ATC/DDD) index (N04-antiparkinsonian drugs, N04A-anticholinergic agents, and N04B-dopaminergic agents); (3) mortality record using codes 332 of the ICD-9 for PD; (4) the minimum basic data set (CMBD) using codes 332 of the ICD-9 for the PD; and (5) death certificates with the G20 code of the ICD-10. Likewise, fully described procedures used for ascertaining PD cases in the EPIC study are previously published by Gallo et al. (28). The diagnosis for each participant was based on a combination of two variables, including the degree of neurologist's expertise and confidence in evaluating the data and quality/amount of the data (29). According to the aforementioned criteria, diagnoses were classified as “definite” (the highest degree of confidence and data quality), “very likely” (high degree of confidence, good/low data quality), “probable” (moderate degree of confidence and great data quality), and “possible” (29). The Navarra center verified all the potential causes.

### Sample Preparation

The straws containing the plasma of each sample were removed from the freezer (−80°C) and slowly thawed on ice. Subsequently, they were opened and transferred to 500 μl Eppendorf tubes, which were kept constantly on ice. They were vortexed for 2 min. A total of 100 μl of plasma were transferred to an Eppendorf tube and 300 μl of a cold mixture (−20°C) of methanol:ethanol (1:1, v/v) were added for deproteinization. After stirring the samples for 1 min, they were incubated on ice for 5 min and vortexed for another 1 min. Samples were centrifuged for 20 min at 13,200 rpm and at 4°C. Finally, 100 μl of supernatant were transferred to a high-performance liquid chromatography (HPLC) vial for analysis.

### Preparation of Blanks and Calibration Samples

Blank samples were prepared in the same way as the plasma samples. A total of 300 μl of methanol:ethanol (1:1, v/v) were added to 500 μl Eppendorf tubes with 100 μl of Milli-Q water. The protocol continued with centrifugation at 13,200 rpm for 20 min. The supernatant was transferred to the HPLC vial for analysis. Calibration samples were prepared from 1,000 ppm stock solutions in methanol:ethanol (1:1, v/v).

### Analytical Setup

This study was conducted using 5 different liquid chromatography-mass spectrometry (LC-MS/MS) methods according to the detectability of the analytes (see Table 1 and Section “Chromatographic Method” in the Supplementary Material). Briefly, one ion-pairing method, two hydrophilic interaction liquid chromatography (HILIC) methods, and two Reversed Phase Liquid Chromatography (RPLC) methods were used combined with tandem mass spectrometry in a triple quadrupole. These methods were partially validated according to the information provided in the Section “Methods Validation” in the Supplementary Material. The analytical performance of the methods has been evaluated by studying the linearity, the repeatability, the intermediate precision, and the sensitivity in terms of limit of detection (LOD) and limit of quantitation (LOQ).

**Table 1:** Corresponding systems, columns, and mobile phases of analytical methods developed in this study.

	Ion-pairing	HILIC A	HILIC B	RPLC A	RPLC B
System	1200 Infinity 6460 QqQ	1260 Infinity II 6470 QqQ	1260 Infinity II 6470 QqQ	1260 Infinity II 6470 QqQ	1260 Infinity II 6470 QqQ
Column	Zorbax Extended C18 (2.1 × 150 mm, 1.8 μm)	XBridgeBEH Amide 2.5 micron (2.1 × 100 mm, 2.5 μm)	XBridgeBEH Amide 2.5 micron (2.1 × 100 mm, 2.5 μm)	Zorbax Eclipse, XDB, C18 (4.6 × 150 mm, 5 μm)	Zorbax Eclipse, XDB, C18 (4.6 × 150 mm, 5 μm)
Mobile phase A	97% water and 3% methanol, 10 mM TBA, 15 mM acetic acid	0.1% Formic acid prepared in water, pH 9 (NH_3_)	5 mM Ammonium formate prepared in water	0.1% Formic acid prepared in water	0.5% Formic acid prepared in water
Mobile phase B	10 mM TBA, 15 mM acetic acid in methanol	0.1% Formic acid prepared in ACN	Acetonitrile	0.1% Formic acid prepared in methanol	0.5% Formic acid prepared in methanol

### Metabolites and Methods

According to the information provided in the introduction, the following analytes were included in this study. Due to the chemical diversity of these metabolites, the analytes were distributed in the 5 analytical methods above-described based on their detectability and selectivity, as shown in Table 2.

**Table 2:** List of studied metabolites and their retention times and transitions in the assigned chromatographic method.

**Metabolic pathway/class**	**Metabolites**	**RT**	**Transitions**	**Methods**
Amino acids and derivatives	Creatinine	1.193	112.0 ⇒ 41.2	Ion-pairing
	Pyroglutamic acid	6.937	128.1 ⇒ 84.0	
Benzoic acids and derivatives	Benzoic acid	15.113	121.1 ⇒ 77.0	
Bile acids	Deoxycholic acid	20.894	391.6 ⇒ 345.2	
Purine metabolism	Hypoxanthine	1.950	135.0 ⇒ 92.0	
	Xanthine	2.523	151.0 ⇒ 108.0	
	Inosine	4.623	267.1 ⇒ 135.0	
	Guanosine	4.896	282.1 ⇒ 150.0	
Fatty acid and dicarboxylic acid metabolism	Palmitic acid	22.446	255.2 ⇒ 256.3	
	Oleic acid	22.559	281.5 ⇒ 282.3	
	Stearic acid	23.252	283.5 ⇒ 283.5	
	Suberic acid	14.951	173.1 ⇒ 111.1	
	Methylmalonic acid	12.097	117.1 ⇒ 71.0	
	Ethylmalonic acid	13.059	131.1 ⇒ 87.1	
Sugars and derivatives	Myoinositol	1.294	239.1 ⇒ 179.0	
TCA cycle	Succinic acid	12.097	117.0 ⇒ 73.1	
	Malic acid	12.806	133.0 ⇒ 115.0	
Tryptophan and kynurenine metabolism	Tryptophan	7.513	203.1 ⇒ 116.0	
	Kynurenic acid	14.589	188.2 ⇒ 144.0	
Amino acids and derivatives	Valine	12.068	118.2 ⇒ 55.1	HILIC A
	Alanine	10.207	90.1 ⇒ 44.1	
Amines	Methylhistamine	11.941	126.2 ⇒ 109.0	
	Trimethylamine	4.409	60.1 ⇒ 44.2	
Purine metabolism	Uric acid	8.763	169.0 ⇒ 141.0	
Amino acids and derivatives	D-methionine	3.298	150.0 ⇒ 61.1	HILIC B
	Serine	4,155	106.1 ⇒ 60.1	
	Threonine	3.893	120.1 ⇒ 74.1	
Tryptophan and kynurenine metabolism	3-hydroxykynurenine	3.439	225.2 ⇒ 208.0	
Alcohols and polyols	Propylene glycol	3.375	77.1 ⇒ 59.1	RPLC A
Dopamine and norepinephrine metabolism	Dopamine	3.482	154.2 ⇒ 137.0	
	3,4-dihydroxyphenylacetic acid	5.345	167.1 ⇒ 123.1	
TCA cycle	Pyruvic acid	2.625	87.0 ⇒ 43.2	
	a-ketoisocaproic acid	5.339	129.1 ⇒ 85.0	
Sugars and others	Threonic acid	2.314	135.1 ⇒ 75.0	
Gamma butyrolactones	Dehydroascorbic acid	6.229	175.1 ⇒ 88.1	RPLC B
Sugars and others	Galactitol	2.227	183.2 ⇒ 129.0	
	Sorbitol	2.227	183.2 ⇒ 129.0	
	D-Gluconic acid	2.249	195.1 ⇒ 129.0	
Tryptophan and kynurenine metabolism	Quinolinic acid	3.807	168.1 ⇒ 78.0	
	Kynurenine	5.312	209.2 ⇒ 192.0	

*RT, retention time expressed in minutes*.

### Data Treatment and Statistical Analysis

After the chromatogram inspection, the obtained data were treated with the MassHunter Quantitative Analysis software (Agilent MassHunter Quantitative Analysis version 10.0) for the determination of the area of each peak. Microsoft Office Excel was used for quantitation and blank subtraction. The *p*-values (the *t*-test or the Wilcoxon/Mann–Whitney *U* test, Microsoft Office Excel, and SPSS, respectively) were calculated for each metabolite. Log_2_FC was calculated according to the following formula:


$$\begin{array}{l} log_{2}FC=log_{2}\;\left(average\;CASES/average\;CONTROLS\right) \end{array}$$

Multivariate statistics were also performed in this study. Supervised orthogonal partial least square-discriminant analysis (OPLS-DA) was performed. Volcano plots plotting variable importance in projection (VIP) in the OPLS-DA model against corrected *p*-values [p(corr), loading values scaled as correlation coefficients values] were generated. Variables with absolute p(corr) lower than 0.3 show a low correlation, while variables between 0.3 and 0.5 show an intermediate correlation. Metabolites with *p*-values < 0.05, VIP score > 1, and p(corr) ≥ 0.3 were considered significant.

The receiver operating characteristic (ROC) curves and the area under the curve (AUC) for the studied metabolites were obtained in Metaboanalyst version 5.0 (30).

## Results

### Demographic Data

This study included 12 case plasma samples from subjects who had developed Parkinson's disease and 21 corresponding control samples. Out of 12 PD subjects, 83.3% were male and 16.7% were female, while in the control group, 80.95% were male and 19.05% were female (Table 3). There was no difference in body mass index (BMI) (*p* = 0.615), age (*p* = 0.782), gender (*p* = 0.865), and smoking status (*p* = 0.632) between patients with PD and healthy control subjects (Table 3). Education information showed that 51.5% of total subjects had primary school completed, 33.3% had none, 6.1% had longer education, i.e., university degree, 6.1% had technical/professional school, and 3% did not specify their education status.

**Table 3:** Demographic characteristics of healthy control subjects and patients with Parkinson's disease (PD) involved in this study.

	**PD patients** *N* = 12	**Control subjects** *N* = 21	**Statistics**
Male (%)	10 (83.33)	17 (80.95)	χ^2^ = 0.029; df = 1; *p* = 0.865
Female (%)	2 (16.66)	4 (19.05)	
Age (years) [median (25th; 75th)]	60.50 (55; 62.50)	60.00 (54.50; 62.50)	U = 118.5; *p* = 0.782
BMI (kg/m^2^) (mean ± SD)	28.45 ± 3.74	29.16 ± 3.91	*t* = −0.508; *p* = 0.615
Smokers (%)	5 (41.7)	7 (33.3)	χ^2^ = 0.229; df = 1; *p* = 0.632
Non-smokers (%)	7 (58.3)	14 (66.7)	

### Targeted Metabolomic Analysis

Out of 40 analytes that were analyzed, seven significant metabolites were observed. Benzoic acid, palmitic acid, oleic acid, stearic acid, myo-inositol, sorbitol, and quinolinic acid were significantly changed in subjects that later developed PD, compared to control subjects. While fatty acids, myo-inositol, and sorbitol were significantly decreased, benzoic acid and quinolinic acid were significantly increased in PD subjects. Despite the assumption that decreased levels of uric acid represent a risk for PD development (21), in this study, the difference in uric acid levels between subjects that later developed PD and healthy control subjects was not observed (Table 4).

**Table 4:** List of analyzed metabolites together with their *p*-values, log_2_FC values, variable importance in projection (VIP), p(corr) scores, corrected *p*-values, and the area under the curve (AUC) scores.

**Metabolites**	***p*** **-value**	**log_**2**_ FC**	**VIP**	**p(corr)**	**q**	**AUC**
**Amino acids and derivatives**						
D-methionine	0.3653	0.10	0.28	0.09	0.6958	0.606
Serine	0.6553	0.03	0.89	0.26	0.8191	0.577
Threonine	0.3898	−0.08	0.63	0.22	0.6779	0.614
Valine	0.3725	−0.06	0.40	0.13	0.6773	0.571
Alanine	0.3450	−0.19	0.90	0.35	0.6900	0.613
Creatinine	0.0619	0.21	1.34	0.31	0.2251	0.688
Pyroglutamic acid	0.2814	−0.13	0.71	0.28	0.6621	0.651
**Tryptophan and kynurenine metabolism**						
Tryptophan	0.9767	0.01	0.11	0.05	1.0017	0.503
Kynurenic acid	0.9877	−0.01	0.68	0.12	0.9877	0.505
3-hydroxykynurenine	0.0789	0.67	1.28	0.38	0.2630	0.701
**Quinolinic acid**	**0.0440**	**0.38**	**1.62**	**0.34**	**0.2514**	**0.728**
Kynurenine	0.1335	0.15	1.43	0.33	0.3814	0.690
**Benzoic acids and derivatives**						
**Benzoic acid**	**0.0385**	**0.31**	**1.30**	**0.34**	**0.2567**	**0.693**
**Bile acids**						
Deoxycholic acid	0.3085	0.24	0.32	0.03	0.6856	0.619
**Purine metabolism**						
Uric acid	0.5773	−0.10	0.81	0.25	0.8553	0.536
Hypoxanthine	0.6508	−0.71	0.72	0.28	0.8397	0.553
Xanthine	0.9366	−0.01	0.36	0.01	1.0704	0.548
Inosine	0.4830	−1.53	0.76	0.26	0.8050	0.582
Guanosine	0.7943	−0.08	0.31	0.00	0.9345	0.503
**Fatty acid and dicarboxylic acid metabolism**						
**Palmitic acid**	**0.0013**	**−1.31**	**1.91**	**0.68**	**0.0520**	**0.893**
**Oleic acid**	**0.0036**	**−1.25**	**1.86**	**0.68**	**0.0720**	**0.854**
**Stearic acid**	**0.0076**	**−1.02**	**1.62**	**0.62**	**0.0760**	**0.792**
Suberic acid	0.0593	0.32	1.03	0.28	0.2636	0.720
Methylmalonic acid	0.2769	0.04	1.30	0.28	0.6923	0.627
Ethylmalonic acid	0.5404	−0.10	0.95	0.32	0.8314	0.571
**Sugars and derivatives**						
Galactitol	0.0570	−0.90	1.21	0.29	0.2850	0.722
**Sorbitol**	**0.0040**	**−2.20**	**1.13**	**0.42**	**0.0533**	**0.836**
D-Gluconic acid	0.5855	−0.10	0.67	0.06	0.8364	0.545
Threonic acid	0.7249	0.07	0.60	0.09	0.8787	0.582
**Myo-inositol**	**0.0211**	**−0.27**	**1.45**	**0.52**	**0.1688**	**0.765**
**TCA cycle**						
Pyruvic acid	0.4958	−0.37	0.40	0.02	0.7933	0.614
a-ketoisocaproic acid	0.6346	0.03	0.76	0.02	0.8461	0.556
Succinic acid	0.6186	0.03	0.95	0.11	0.8532	0.558
Malic acid	0.0936	0.11	1.08	0.14	0.2880	0.656
**Amines**						
Methylhistamine	0.9611	−0.03	0.15	0.06	1.0390	0.506
Trimethylamine	0.3191	−0.18	1.17	0.45	0.6718	0.610
**Alcohols and polyols**						
Propylene glycol	0.0607	−0.49	1.50	0.40	0.2428	0.683
**Dopamine and norepinephrine metabolism**						
Dopamine	0.9609	−0.06	0.66	0.21	1.0677	0.505
3,4-dihydroxyphenylacetic acid	0.2210	−0.23	0.78	0.10	0.5893	0.643
**Gamma butyrolactones**						
Dehydroascorbic acid	0.9639	0.06	0.33	0.10	1.0146	0.505

*p-value - level of significant obtained with t-test or Mann-Whitney U test; log_2_ FC - was calculated according to the following formula: log_2_FC - log_2_ (averageCASES/averageCONTROLS); VIP, variable importance in the projection in OPLS-DA model; p(corr), loading values scaled as correlation coefficients values in OPLS-DA model; q, Benjamini-Hochberg (FDR, false discovery rate) correction; AUC, area under the curve*.

*Significant metabolites found in this study were bolded*.

**Figure 1:**
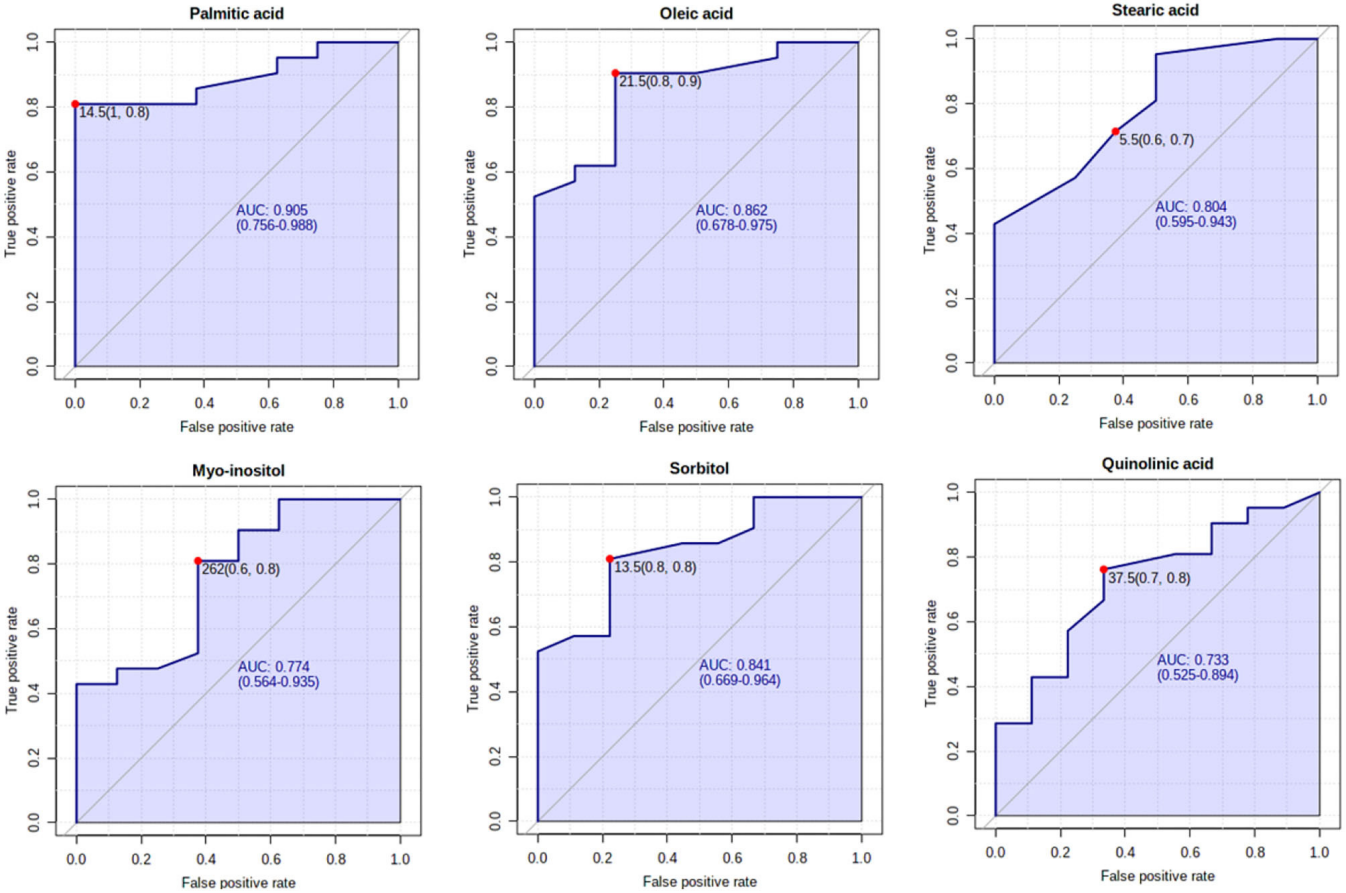
The receiver operating characteristic (ROC) curves of significantly different metabolites between Parkinson's disease (PD) cases and control subjects, with the area under the curve (AUC) > 0.7.

**Figure 2:**
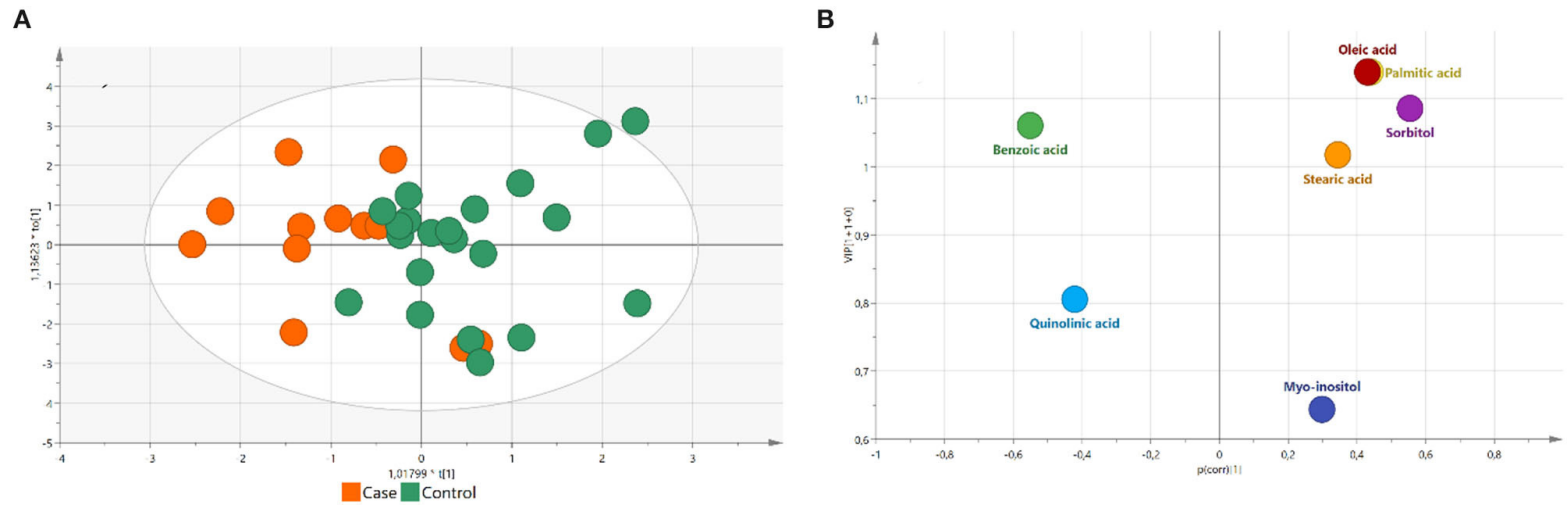
**(A)** Orthogonal partial least square-discriminant analysis (OPLS-DA) plot for significantly different compounds between PD cases and control subjects (R2X = 0.563, R2Y = 0.402, and Q2 = 0.165). Cases are marked as orange and controls are marked as green; **(B)** Volcano plot plotting VIP in OPLS-DA model against p[corr] value for significantly different compounds between PD cases and control subjects. Colored according to identifiers.

The ROC curves of significant metabolites with the AUC > 0.7 (palmitic acid, oleic acid, stearic acid, myo-inositol, sorbitol, and quinolinic acid) are shown in Figure 1. OPLS-DA and volcano plots for seven significant compounds are given in Figure 2.

The analytical parameters for significant compounds are shown in Table 4. All the significant compounds showed good repeatability and intermediate precision, with a coefficient of variation > 0.99 (Table 5). The analytical performance of the five chromatographic methods is shown in the Supplementary Material in the Section “Method Validation.”

**Table 5:** The analytical parameters of significantly different metabolites between PD cases and control subjects.

Metabolites	Transition	Linearity (ppm)	R	Repeatability (%RSD)	Intermediate precision (%RSD)	LOD	LOQ
Palmitic acid	255.2⇒256.3	1–25	0.9960	5.4	26.0	475.1	1,583.7
Oleic acid	281.5⇒282.3	1–25	1.0000	6.5	17.5	247.9	826.5
Stearic acid	283.5⇒283.5	1–25	0.9998	9.3	27.7	655.3	2,184.4
Benzoic acid	121.1⇒77.0	0.25–2	0.9996	2.4	6.3	80.1	267.0
Myoinositol	239.1⇒179.0	0.025–0.5	0.9970	1.3	6.0	23.6	78.6
Sorbitol	183.2⇒129.0	0.002–0.5	0.9990	1.6	10.1	1.6	5.2
Quinolinic acid	168.1⇒78.0	0.002–1	0.9999	0.9	19.0	1.7	5.7

^*^linearity expressed in ppm; R, coefficient of variation; repeatability and intermediate precision expressed as %RSD; LOD, limit of detection; LOQ, limit od quantitation.

## Discussion

Parkinson's disease is a complex, heterogeneous neurodegenerative disorder with an expected rise in prevalence up to 9 million in 2030 (31). Thus, an increasing number of patients with PD might cause a high financial and social burden (32). Clinical diagnosis of PD is usually established when first parkinsonian symptoms appear and when there is already a significant dopaminergic loss (10). Therefore, due to the lack of biomarkers for early diagnosis of PD, targeting metabolites that could be involved in PD development and progression might improve therapeutic efficiency and provide a better understanding of the underlying molecular mechanisms that lead to PD development (31), as well as improving the quality life of patients and relieve pressure on medical services.

### Altered Metabolites

Out of the 40 studied compounds related to PD, seven statistically significant (*p* < 0.05) metabolites have been observed in PD subjects compared to healthy control subjects. Benzoic acid, palmitic acid, oleic acid, stearic acid, myo-inositol, sorbitol, and quinolinic acid were significantly changed in PD subjects. Altered levels of sugar alcohols might indicate alterations in the sugar metabolism. It is assumed that increased glucose levels could overcome glycolysis capacity, which causes conversion of glucose to sorbitol. Another altered metabolite that was significantly altered is myo-inositol, which also plays an important role in sugar metabolism. It is known that altered levels of myo-inositol, together with altered levels of sorbitol, might be associated with changes in glucose metabolism and glycolysis (9). Alterations of glycolysis and sugar metabolism imply on potential involvement of metabolic pathways that participate in the energy production in pathogenesis of PD (11). Besides, malabsorption of sorbitol might be associated with gastrointestinal dysfunction. Bacterial overgrowth causes changes in the gut mucosa that leads to sugar malabsorption (33). Bacterial overgrowth, as well as other gastrointestinal dysfunctions, is common among subjects with PD. Up to 80% of patients with PD show some signs of gastrointestinal impairments, which usually appear in the early stage of PD (34). Altered levels of galactitol and sorbitol in subjects with PD have been observed in other studies as well (11). Decreased levels of myo-inositol, galactitol, and sorbitol were reported in this study, while Ahmad et al. (9) showed decreased levels of galactitol but increased levels of sorbitol and myo-inositol. However, inconsistent results might indicate a lack of possible association between PD development and impairments of sugar metabolism and energy production in the early stage of Parkinson's disease or even before the illness has appeared. Furthermore, decreased levels of fatty acids in subjects with PD have been observed in this study. Palmitic, oleic, and stearic acids were significantly decreased. Such a result is in correspondence with other studies that found a reduction in fatty acids levels in patients with PD (4). Havelund et al. (4) found, among others, decreased levels of palmitic, oleic, and stearic acids in subjects with PD. The metabolism of fatty acids has repeatedly been associated to the development and pathogenesis of PD. Changes in fatty acids might be associated with mitochondrial dysfunction (4, 5, 35), neuroinflammation (14), alterations in apoptotic signaling (36), as well as with oxidative stress (13). Even more, these findings agree with recent discoveries published by our group (29) in which a different cohort of a similar number of subjects was used for the blind discovery of prognostic biomarkers of PD using untargeted metabolomics. In this study, several fatty acids, including the ones reported here, were also found statistically decreased. This supports that changes in the metabolism of fatty acid could help to understand the progression of PD and the involved species could be string candidates as biomarkers for early diagnosis of the disease. Quinolinic acid is an intermediate compound in the tryptophan-kynurenine metabolic pathway, which has already been associated with PD development (8). Kynurenine is the main intermediate compound and it can be metabolized in two ways to kynurenic acid, which acts as a neuroprotective agent or to 3-hydroxykynurenine and quinolinic acid, which are neurotoxic. Increased levels of quinolinic acid cause neuron excitation by activation of N-methyl-D-aspartate (NMDA) receptors, which consequently lead to excitotoxicity, increased inflammation, and eventually to neuronal death (37). It is known that degeneration of dopaminergic neurons in the substantia nigra in Parkinson's disease is a result of excitotoxicity. Recent studies (6, 38) showed that altered kynurenine pathways and their metabolites are present in plasma and cerebrospinal fluid, respectively, in subjects with PD. This is in correspondence with our finding of altered levels of quinolinic acid. In this study, increased concentration of quinolinic and 3-hydroxykynurenine has been found in patients with PD, compared with healthy control subjects, while similar findings were as well found by Heilman et al. (7). However, 3-hydroxykynurenine was not significantly increased, although it showed a trend in subjects with PD. It is assumed that alterations in these metabolites are associated with the severity of PD symptoms (7). Dysfunction of tryptophan and the kynurenine pathway might result in increased oxidative stress, as well as neuroinflammation that would lead to neurodegenerative processes characteristic for PD. Therefore, increased levels of quinolinic acid in the subjects that later developed PD might indicate alterations in the kynurenine pathway in the early stage, before first PD symptoms appear and might represent potential biomarker for early diagnosis or lead to development of therapeutic approach that could target kynurenine to 3-hydroxykynurenine conversion (8).

Globally, the metabolites that have been found to be significantly altered in this study are related to mitochondrial dysfunction, oxidative stress, and the mechanisms of energy production. Considering that all the volunteers enrolled in this study were healthy at the time of sample collection, these findings might imply that these processes begin to be affected before PD shows any symptoms. This information is of great relevance for a disease, such as PD, in which the definition of a metabolite panel that can be used for the early diagnosis of the disease has been sought for decades.

### European Prospective Investigation Into Cancer and Nutrition Samples for Finding Biomarkers for the Early Diagnosis of Parkinson's Disease

**Figure 3:**
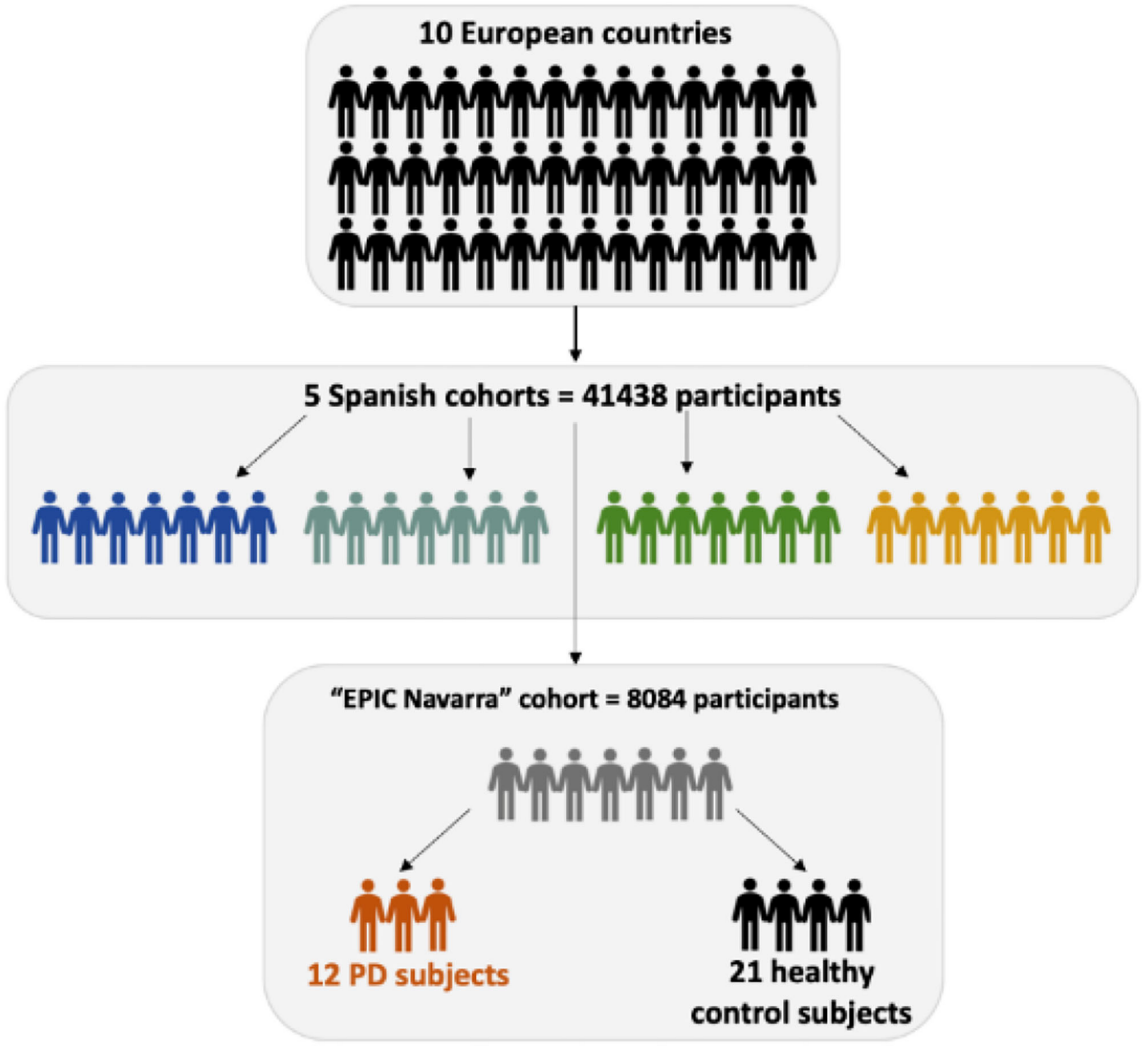
Schematic structure of the European Prospective Investigation into Cancer and Nutrition (EPIC) study and the samples used in study.

We are aware of the limitations of this study in terms of the sample size and the need for validation in larger studies. However, we consider the most important aspect of this study is the use of true reliable samples that allow for well-founded biomarkers for the early diagnosis of PD. The samples used in this study are of invaluable importance. They were obtained from the large prospective EPIC study, in which volunteers were followed-up for years, not just for cancer events, which were the main objective of this study, but for the development of many other chronic diseases, such as cardiovascular disease, type 2 diabetes, PD, and also mortality or even healthy aging. We want to highlight that the samples included in this study, which include plasma, serum, leukocytes, and erythrocytes, are searchable and researchers can apply for their collection and use in their research studies. This study used samples from the EPIC cohort in Navarra (Spain), which enrolled 8,084 participants (Figure 3). The two other EPIC-Spain cohorts have undergone the ascertainment of PD cases, for a total of 25,016 participants and 69 PD cases occurred during over 15 years of follow-up. However, Spain has a total of 5 cohorts, counting up to 41,438 participants and it is just one of the 10 participating countries. All this, considered together, provides multiple options worth of investigation.

As mentioned, a recently published article (29) on samples from the EPIC-Spain cohort identified several altered metabolites in subjects that subsequently developed PD. The results obtained after untargeted analysis showed that mostly fatty acids, as well their derivatives have been altered in PD. This is in correspondence with this targeted study in which we confirmed previously published results. Levels of palmitic, oleic, and stearic acids were decreased in both of these studies, indicating possible role of aforementioned fatty acids as potential prognostic biomarkers for PD (29). One of the premises of this study was that some of the metabolites that are altered in conditions of established PD might also be altered before and, therefore, could be used as biomarkers for the early diagnosis of PD. A total of 40 metabolites were selected for this study because they were reported in cases of PD. Among them, only seven metabolites are statistically changed in our samples. This does not exclude the possibility that other metabolic pathways could be altered before the disease is stabilized and other studies of different natures should be performed, which aim for the blind discovery of differential metabolites. Our results prove that the changes in the metabolic profile vary with the time over PD is developed. This is not surprising and has been the main reason why the discovery of prognostic biomarkers for PD is still under study. For this reason, it is critical to go as much as possible back in time before the patients develop symptoms, indicating that the disease has already been established. In this regard, the samples from the EPIC study represent a great opportunity for the evaluation of panels of metabolites that might be altered in subjects before they developed a disease and to propose them as true prognostic biomarkers of PD.

## Conclusion

The greatest strength of this study lays in possibilities of the EPIC samples for finding biomarkers for the early diagnosis of PD. The fact that all the donors of these samples were healthy at the time of the sample collection makes these samples of trusted value for such a purpose. We consider the samples stored in the different cohorts of the EPIC study can also be used for many other purposes, since massive data, such as life conditions, development of diseases, and time of death or survival, are stored for all the participants, who were followed-up for 15 years, which is of an extreme importance for clinicians and future study. These samples can be searched in order to find healthy donors for a particular disease, who developed that particular disease over the time. In our opinion, the possibilities of these samples are almost endless and these samples are of extreme value for those researchers looking for small changes that occur before a disease is stabilized. This study provided adequate analytical techniques for the metabolites to be studied, which has ensured to have a wide panel of metabolites to be evaluated. We have confirmed that some of the metabolites that are altered in PD seem to be also altered before the disease shows any symptoms. These significant metabolites indicate possible association of mitochondrial dysfunction, alteration in energy metabolism and tryptophan metabolism, as well as oxidative stress as the molecular mechanisms that could lead to the development and progression of a complex neurodegenerative disorder, such as PD.

However, certain limitations should be addressed. Limitations of this study include small sample size and the need for confirmation and validation of these findings in bigger studies, while other biological pathways should be investigated for finding reliable biomarkers for the diagnosis of PD. Due to large number of analyzed metabolites, correction of *p*-value has been performed. However, after the Benjamini–Hochberg correction of *p*-value, significance for all the metabolites was lost. Significant metabolites after adjusting *p*-value are still showing trend in patients with PD, especially fatty acids and sorbitol. Since the EPIC-Navarra is relatively homogeneous cohort, future studies should include larger number of samples, not only from Spain, but from other independent cohorts or centers in Europe that were part of the EPIC study, to validate and replicate metabolites that are considered as potential prognostic biomarkers of PD. Besides the fact that most of the studied metabolites were not significant in this study, it indicates that the metabolic profile evolves over the development of the disease. This point out that not all the biomarkers related to PD can be used as prognostic biomarkers and reliable samples must be used to face the challenge of prognosis in PD.

## Data Availability Statement

The original contributions presented in the study are included in the article/Supplementary Material, further inquiries can be directed to the corresponding author/s.

## Ethics Statement

This study was approved by the Ethics Committee for clinical research of the Basque Country PI2017031 and written informed consent was obtained from pre-PD subjects and controls according to approved protocols.

## Author Contributions

MK and JS were involved in sample preparation, sample analysis, data treatment, statistical analysis, preparation, and review of the manuscript. AB, PA, JH, EAr, and CB conceived the study and design study. JS, CB, EAr, JH, JI, PA, AB, AV-A, ME, and MC collected data. MK, JS, CB, AB, EAr, EAb, and JH performed data interpretation. All authors have contributed to the article and approved the submitted version of the manuscript.

## Funding

The EPIC study received financial support from the International Agency for Research on Cancer (AEP/93/06), the European Commission (SO-97-200302-05F02 and SP23-CT-2005-006438), the Health Research Fund (FIS) of the Spanish Ministry of Health, the Red Temática de Investigación Cooperativa de Centros de Cáncer (RTICCC C03/10 and RD06/0020), the Consortium for Biomedical Research in Epidemiology and Public Health (CIBERESP), the participating Regional Governments of Andalusia, Basque Country, Murcia and Navarra, and the Catalan Institute of Oncology (ICO). This study was furthermore supported by the Ministry of Health of the Basque Government, Exp 201611098.

## Conflict of Interest

The authors declare that the research was conducted in the absence of any commercial or financial relationships that could be construed as a potential conflict of interest.

## Publisher's Note

All claims expressed in this article are solely those of the authors and do not necessarily represent those of their affiliated organizations, or those of the publisher, the editors and the reviewers. Any product that may be evaluated in this article, or claim that may be made by its manufacturer, is not guaranteed or endorsed by the publisher.
