# Supplementary material for: A Set of Reliable Samples for the Study of Biomarkers for the Early Diagnosis of Parkinson's Disease
Source: Front Neurol. 2022 May 30;13:844841. doi: 10.3389/fneur.2022.844841 (PMC9189395; doi:10.3389/fneur.2022.844841)
Supplement: Supplementary file 1 [file Table_1.DOCX]

Supplementary Material

# Chromatographic methods

## Ion - pairing reverse phase chromatography

The analysis was carried out in an Agilent 1200 Infinity (Agilent Technologies, Palo Alto, Calif.) using a 1290 Infinity Binary Pump and a 1260 Infinity Quaternary Pump. The LC system was coupled to an Agilent 6460 triple quadrupole mass spectrometer (MS) using an electrospray ionization (ESI) interface working in MRM mode. The analytical setup for the chromatography was previously described by Agilent Technologies, Inc. (MassHunter Metabolomics dMRM Database and Method, Analysis guide, Revision B, Agilent Technologies, Inc. 2016.; MassHunter Metabolomics Dynamic MRM Database and Method, Method Setup Guide, Revision B, Agilent Technologies, Inc. 2016). This method uses tributylamine as ion-pair reagent, with buffer A composed of 97% water and 3% methanol, 10 mM tributylamine, 15 mM glacial acetic acid and buffer B composed of 10 mM tributylamine, 15 mM glacial acetic acid, prepared in methanol. The flow was set at 0.25 mL/min, with maximum pressure set at 1050 bars. The method described by Agilent uses a 1290 Infinity II LC Quaternary Pump and a 6-port/2-position valve for the gradient chromatography, in a first stage, for the removal of contaminants at the column head for each and every analytical run in back-flush mode with acetonitrile, in a second stage, and for the column regeneration with buffer A, in a third stage. The set-up in our laboratory was different, having a binary, a quaternary pumps and a 10-port/2-position valve. Therefore, the hardware connection and the chromatography had to be modified in order to keep equivalent separation, cleaning and regeneration stages to those proposed in the original method. The analysis started with 0 % of the mobile phase B, which has been gradually increased up to 99 % at minute 20. Such conditions were maintained until minute 24.10, when % of mobile phase B was decreased to starting conditions. All the samples were analyzed in negative ESI mode. The capillary voltage was set to 2000 V for negative ionization mode. Gas nebulizer was maintained at 45 psi, while drying gas flow rate was 13 L/min at 150 ° C Samples were injected randomly with an injection volume set to 5 µL and the autosampler was kept at 4ºC.

**Hydrophilic interaction chromatography (methods A and B)**

The analysis was carried out in an Agilent 1290 Infinity (Agilent Technologies, Palo Alto, Calif.) using a 1290 Infinity Binary Pump. The LC system was coupled to an Agilent 6470 triple quadrupole mass spectrometer (MS), using an ESI interface, working in MRM mode. For the HILIC A method, the mobile phase A consisted of 0.1% formic acid prepared in water, pH 9, while for mobile phase B was 0.1% formic acid prepared in acetonitrile. For HILIC B method, mobile phase A was 5mM ammonium formate prepared in water was used, while mobile phase B was pure acetonitrile. For the gradient chromatography, the flow was set at 0.6 mL/min for HILIC A or 0.8 mL/min for HILIC B method. The analysis for HILIC A method started with 93 % of the mobile phase B, which is gradually decreased to 50 % at minute 15. From the minute 15.50 up to minute 20 starting conditions were maintained. All the samples were analyzed in positive and negative ESI mode. The capillary voltage was set to 4000 V for positive and 3000 V for negative ionization mode. Gas nebulizer was maintained at 60 psi, while drying gas flow rate was 5 L/min at 300 ° C. The elution conditions for HILIC B method started with 50 % of the mobile phase B and increased to 95% in minute 7. These conditions were retained until minute 10. All the samples were analyzed in positive ESI mode. The capillary voltage was set to 3500 V, gas nebulizer was maintained at 45 psi, while drying gas flow rate was 8 L/min at 250 ° C. Five microliters of the prepared samples was injected onto a XBridgeTM BEH Amide column (2.5 µm, 100 × 2.1 mm, Waters, Dublin, Ireland), while the autosampler was kept at 4ºC.

**Reversed - phase liquid chromatography (methods A and B)**

RPLC A and B methods were carried out in the same system as for the HILIC methods. The mobile phases just differed in the amount of formic acid. As mobile phase A for RPLC A method, 0.1% formic acid prepared in water was used, while mobile phase B was 0.1% formic acid prepared in methanol. RPLC B method used the same solvents in which 0.5 % of formic acid was added. The flow was set at 0.6 mL/min. The analysis for RPLC A method started with 1% of the mobile phase B and then increased to the 95% of B in a time period of 2 min (0-2 min), after which it has been again decreased to the 1% of B (3-10 min). The elution conditions for RPLC B method had same starting conditions as RPLC A method, with exception that 95% of mobile phase B lasted one minute longer than in RPLC A method. All the samples were analyzed in positive and negative ESI mode. The capillary voltage was set to 3500 V for positive and 3000 V for negative ionization mode. Gas nebulizer was maintained at 45 psi, while drying gas flow rate was 8 L/min at 250 ° C. The column temperature was maintained at 45°C. Samples were injected randomly with an injection volume set to 5 µL onto Zorbax Eclipse, XDB, C18 (4.6 x 150 mm, 5 µm) column, while the autosampler was kept at 4ºC.

# Methods validation

Five experimental methods with different conditions, mobile phases and columns were studied in order to get the best separation and detection of selected analytes. The analytical performance, repeatability, intermediate precision, limit of detection (LOD), limit of quantitation (LOQ) and linearity were evaluated for each analyte for each method.

The LOD for each compound was calculated by plotting 3 x standard deviation of the response of the analyte at a concentration of approximately the LOQ in the calibration curve:

LOD = 3 x SD / s

The LOQ was calculated as follows, by plotting 10x standard deviation of the response in the calibration curve:

LOQ = 10 x SD / s

The precision was estimated in terms of repeatability and intermediate precision. Repeatability was calculated in five repeated analyses at the same concentrations while intermediate precision was calculated at two concentrations, 0.1 μg/ml and 1 μg/ml in five non-consecutive days over one week.

The linear working range was calculated from 10 different concentrations of the analytes between 1 ppb and 2000 ppb. These concentrations were: 1 ppb, 5 ppb, 10 ppb, 25 ppb, 50 ppb, 100 ppb, 250 ppb, 500 ppb, 1000 ppb and 2000 ppb, while for fatty acids linear working range was calculated from 4 different concentrations of the analytes: 1000 ppb, 5000 ppb, 10000 ppb and 25000 ppb. R values were calculated according to these concentrations from the calibration curve.

*a) Ion-pairing chromatography:* Nineteen metabolites evaluated with this method showed good repeatability and intermediate precision, while R for each analyte was more than 0.99. Linear working range differed between different metabolites, while fatty acids showed bigger linear working range than other studied metabolites, from 1 to 25 ppm (Table S1). The lowest limit of detection, as well as the limit of quantitation, were observed for ethylmalonic and kynurenic acid (Table S1).

*b) HILIC method A:* Five metabolites were included in the HILIC method A. For each of them R was higher than 0.98. Good repeatability and intermediate precision were estimated for most of the compounds. Linearity for most of the analytes was between 0.5 ppm - 2.5 ppm, with exception of uric acid that showed the biggest linear working range, from 10 ppm – 25 ppm. The smallest limit of detection and quantitation were observed for amines, methylhistamine and trimethylamine (Table S2).

*c) HILIC, method B:* Four metabolites were analyzed using HILIC method B. Analytes showed very good repeatability (less than 1% RSD) and intermediate precision. Coefficient of correlation was higher than 0.99. Linearity for methionine and kynurenine was between 0.005 ppm - 1 ppm, while serine and threonine had linear working range between 0.5 ppm - 2 ppm. Limit of detection and limit of quantitation for serine, threonine and 3-hydroxykynurenine was around 10 ppb for LOD and 30 ppb for LOQ, while methionine showed a slightly larger LOD and LOQ (Table S3).

*d) RPLC method A:* Six metabolites were analyzed by RPLC method A. Linear working ranges varied among the different metabolites. Coefficient of correlation for each analyte was always higher than 0.99. Good repeatability and intermediate precision were observed, while threonic acid showed the best repeatability and intermediate precision. The lowest LOD and LOQ were observed for α-ketoisocaproic acid, while the biggest LOD and LOQ were calculated for pyruvic acid (Table S4).

*e) RPLC method B:* Same as in previous RPLC method, six metabolites were analyzed. For each of them R was higher than 0.99, while good repeatability and intermediate precision was estimated for all analytes. Linearity for most of analytes was between 0.002 ppm and 1 ppm. The lowest LOD and LOQ were calculated for kynurenine, with LOD at 0.2 ppb and LOQ at 0.7 ppb, which is the lowest precision among all analyzed compounds in this study (Table S5).

Figure S1 shows the chromatograms for the metabolites studied with their respective assigned methods. This figure shows the good sensitivity achieved in the analysis throughout the chromatographic separation and the MRM analysis.

**Table S1.** Metabolites and their analytical parameters used in ion-pairing chromatography.

| ION PAIRING | Quantitative transitions | Qualitative transitions | Linearity (ppm) | R | Repeatability (%RSD) | Intermediate precision (%RSD) | LOD | LOQ |
| --- | --- | --- | --- | --- | --- | --- | --- | --- |
| Myoinositol | 239.1 🡪 179.0 | 239.1 🡪 71.0 | 0.025 - 0.5 | 0.9970 | 1.3 | 6.0 | 23.6 | 78.6 |
| Creatinine | 112.0 🡪 41.2 | 112.0 🡪 68.1 | 0.1 - 2 | 0.9960 | 1.9 | 7.3 | 56.9 | 189.7 |
| Hypoxanthine | 135.0 🡪 92.0 | 135.0 🡪 65.1 | 0.25 - 2 | 0.9970 | 1.5 | 5.6 | 29.2 | 97.4 |
| Xanthine | 151.0 🡪 108.0 | 151.0 🡪 42.2 | 0.01 - 1 | 0.9970 | 1.3 | 6.0 | 31.7 | 105.8 |
| Inosine | 267.1 🡪 135.0 | 267.1 🡪 108.0 | 0.1 - 2 | 0.9997 | 3.6 | 5.2 | 30.2 | 100.7 |
| Guanosine | 282.1 🡪 150.0 | 282.1 🡪 132.9 | 0.01 - 1 | 0.9970 | 1.0 | 17.2 | 179.3 | 597.9 |
| Pyroglutamic acid | 128.1 🡪 84.0 | 128.1 🡪 128.1 | 0.025 - 0.5 | 0.9980 | 0.4 | 9.6 | 14.3 | 47.8 |
| Tryptophan | 203.1 🡪 116.0 | 203.1 🡪 74.1 | 0.05 - 2 | 0.9995 | 0.5 | 3.2 | 11.7 | 39.0 |
| Methylmalonic acid | 117.1 🡪 71.0 | 117.1 🡪 55.0 | 0.05 - 1 | 0.9995 | 2.5 | 11.3 | 18.2 | 60.6 |
| Succinic acid | 117.0 🡪 73.1 | 117.0 🡪 99.0 | 0.5 - 2 | 0.9930 | 3.2 | 17.1 | 48.9 | 161.9 |
| Malic acid | 133.0 🡪 115.0 | 133.0 🡪 71.1 | 0.05 - 1 | 0.9980 | 3.4 | 16.6 | 68.9 | 229.6 |
| Ethylmalonic acid | 131.1 🡪 87.1 | 131.1 🡪 69.1 | 0.002 - 1 | 0.9980 | 1.3 | 10.4 | 3.3 | 11.1 |
| Suberic acid | 173.1 🡪 111.1 | 173.1 🡪 83.1 | 0.025 - 0.5 | 0.9980 | 1.4 | 9.7 | 48.0 | 160.0 |
| Kynurenic acid | 188.2 🡪 144.0 | 188.2 🡪 188.2 | 0.001 - 0.5 | 0.9970 | 1.4 | 5.1 | 0.9 | 3.1 |
| Benzoic acid | 121.1 🡪 77.0 | 121.1 🡪 121.1 | 0.25 - 2 | 0.9996 | 2.4 | 6.3 | 80.1 | 267.0 |
| Deoxycholic acid | 391.6 🡪 345.2 | 391.6 🡪 391.6 | 0.025 - 2 | 0.9960 | 3.0 | 21.0 | 214.7 | 715.6 |
| Palmitic acid | 255.2 🡪 256.3 | 255.2 🡪 255.2 | 1 - 25 | 0.9960 | 5.4 | 26.0 | 475.1 | 1583.7 |
| Oleic acid | 281.5 🡪 282.3 | 281.5 🡪 281.5 | 1 - 25 | 1.000 | 6.5 | 17.5 | 247.9 | 826.5 |
| Stearic acid | 283.5 🡪 283.5 | 283.5 🡪 283.5 | 1 - 25 | 0.9998 | 9.3 | 27.7 | 655.3 | 2184.4 |

**Table S2.** Metabolites and their analytical parameters used in HILIC A.

| HILIC A | Quantitative transitions | Qualitative transitions | Linearity (ppm) | R | Repeatability (%RSD) | Intermediate precision (%RSD) | LOD | LOQ |
| --- | --- | --- | --- | --- | --- | --- | --- | --- |
| Methylhistamine | 126.2 🡪 109.0 | 126.2 🡪 68.1 | 0.5 - 2.5 | 0.996 | 0.2 | 1.9 | 0.2 | 0.6 |
| Uric acid | 169.0 🡪 141.0 | 169.0 🡪 43.1 | 10 - 50 | 0.993 | 2.5 | 15.3 | 36.4 | 121.3 |
| Trimethylamine | 60.1 🡪 44.2 | 60.1 🡪 45.2 | 0.5 - 2.5 | 0.98 | 0.4 | 7.3 | 1.8 | 6.2 |
| Valine | 118.2 🡪 55.1 | 118.2 🡪 72.1 | 0.5 - 2.5 | 0.997 | 0.5 | 4.3 | 6.7 | 22.4 |
| Alanine | 90.1 🡪 44.1 | 90.1 🡪 27.2 | 0.5 - 2.5 | 0.997 | 0.2 | 6.7 | 15.4 | 51.4 |

**Table S3.** Metabolites and their analytical parameters used in HILIC B.

| HILIC B | Quantitative transitions | Qualitative transitions | Linearity (ppm) | R | Repeatability (%RSD) | Intermediate precision (%RSD) | LOD | LOQ |
| --- | --- | --- | --- | --- | --- | --- | --- | --- |
| D-methionine | 150.0 🡪 61.1 | 150.0 🡪 56.1 | 0.005 - 2 | 0.997 | 0.4 | 2.2 | 22.0 | 73.3 |
| 3-hydroxykynurenine | 225.2 🡪 208.0 | 225.2 🡪 162.0 | 0.005 - 1 | 0.9997 | 0.6 | 8.9 | 7.8 | 26.0 |
| Serine | 106.1 🡪 60.1 | 106.1 🡪 42.1 | 0.5 - 2 | 0.9995 | 0.8 | 22.2 | 10.8 | 36.0 |
| Threonine | 120.1 🡪 74.1 | 120.1 🡪 56.1 | 0.5 - 2 | 0.9994 | 0.5 | 18.3 | 7.7 | 25.7 |

**Table S4.** Metabolites and their analytical parameters used in RPLC A.

| RPLC A | Quantitative transitions | Qualitative transitions | Linearity (ppm) | R | Repeatability (%RSD) | Intermediate precision (%RSD) | LOD | LOQ |
| --- | --- | --- | --- | --- | --- | --- | --- | --- |
| Threonic acid | 135.1 🡪 75.0 | 135.1 🡪 89.0 | 0.01 - 1 | 0.9995 | 1.4 | 10.9 | 10.4 | 34.8 |
| Propylene glycol | 77.1 🡪 59.1 | 77.1 🡪 77.1 | 0.25 - 1 | 0.99 | 1.6 | 32.4 | 49.7 | 165.8 |
| Dopamine | 154.2 🡪 137.0 | 154.2 🡪 91.0 | 0.005 - 0.025 | 0.9998 | 3.3 | 26.8 | 90.1 | 300.3 |
| Pyruvic acid | 87.0 🡪 43.2 | 87.0 🡪 43.2 | 0.25 - 2 | 0.995 | 3.7 | 19.4 | 147.5 | 491.7 |
| 3.4-dihydroxyphenylacetic acid | 167.1🡪123.1 | 167.1🡪123.1 | 0.001 - 0.01 | 0.9998 | 1.5 | 21.8 | 9.9 | 32.9 |
| α-ketoisocaproic acid | 129.1 🡪 85.0 | 129.1 🡪 129.1 | 0.5 - 2 | 0.9992 | 1.9 | 16.6 | 3.6 | 12.1 |

**Table S5.** Metabolites and their analytical parameters used in RPLC B.

| RPLC B | Quantitative transitions | Qualitative transitions | Linearity (ppm) | R | Repeatability (%RSD) | Intermediate precision (%RSD) | LOD | LOQ |
| --- | --- | --- | --- | --- | --- | --- | --- | --- |
| Galactitol | 183.2 🡪 129.0 | 183.2 🡪 57.1 | 0.01 - 0.5 | 0.9998 | 1.6 | 10.1 | 1.7 | 5.7 |
| D-Gluconic acid | 195.1 🡪 129.0 | 195.1 🡪 72.0 | 0.025 - 2 | 0.998 | 0.4 | 13.1 | 13.7 | 45.5 |
| Sorbitol | 183.2 🡪 129.0 | 183.2 🡪 69.0 | 0.002 - 0.5 | 0.999 | 1.6 | 10.1 | 1.6 | 5.2 |
| Quinolinic acid | 168.1 🡪 78.0 | 168.1 🡪 149.9 | 0.002 - 1 | 0.99995 | 0.9 | 19.0 | 1.7 | 5.7 |
| Kynurenine | 209.2 🡪 192.0 | 209.2 🡪 94.0 | 0.002 - 1 | 0.99995 | 0.4 | 12.0 | 0.2 | 0.7 |
| Dehydroascorbic acid | 175.1 🡪 88.1 | 175.1 🡪 46.2 | 0.25 - 1 | 0.9992 | 1.5 | 4.4 | 149.7 | 499.0 |

**
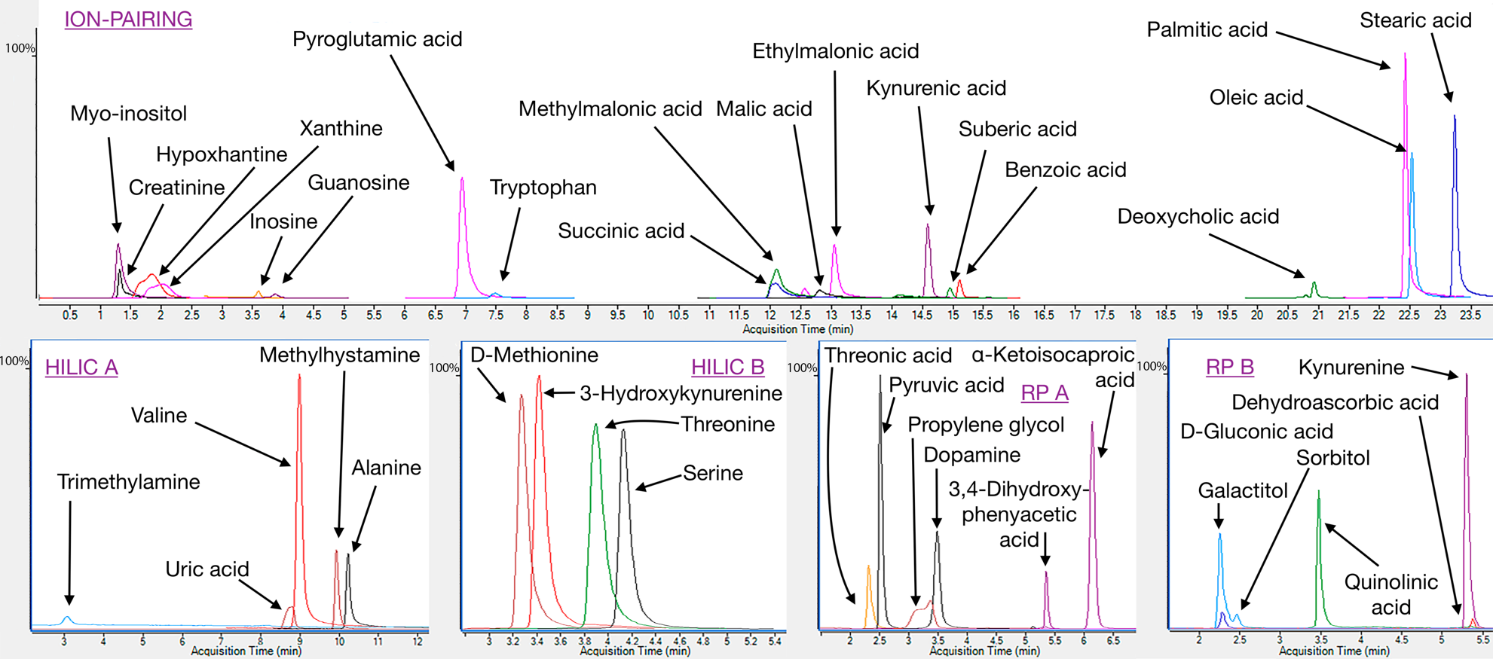
**

**Supplementary Figure 1.** Chromatographic peaks of metabolites studied with their respective assigned methods.
